# Risk of Myelopathy Following Second Local Treatment after Initial Irradiation of Spine Metastasis

**DOI:** 10.3390/diagnostics13020175

**Published:** 2023-01-04

**Authors:** Laurentia Gales, Diana Mitrea, Bogdan Chivu, Adrian Radu, Silvia Bocai, Remus Stoica, Andrei Dicianu, Radu Mitrica, Oana Trifanescu, Rodica Anghel, Luiza Serbanescu

**Affiliations:** 1 Department of Oncology, “Carol Davila” University of Medicine & Pharmacy, 020021 Bucharest, Romania; 2 Department of Oncology, “Prof. Dr. Alexandru Trestioreanu” Institute of Oncology, 022328 Bucharest, Romania; 3 Department of Radiotherapy, “Prof. Dr. Alexandru Trestioreanu” Institute of Oncology, 022328 Bucharest, Romania; 4 Department of Radiotherapy, Centrul Oncologic Sanador, 010991 Bucharest, Romania; 5 Department of Radiotherapy, Clinical Emergency County Hospital, 200642 Craiova, Romania

**Keywords:** myelopathy, SBRT, spine metastasis, reirradiation, Lhermitte, palliation, spinal cord compression

## Abstract

Metastatic lesions of the spine occur in up to 40% of cancer patients and are a frequent source of pain and neurologic deficit due to cord compression. Palliative radiotherapy is the main first-intent local treatment in the form of single-fraction radiotherapy or fractionated courses. Reirradiation is a viable option for inoperable patients where spinal decompression is needed but with an increased risk of radiation-induced myelopathy (RM) and subsequent neurologic damage. This review summarizes reported data on local treatment options after initial irradiation in patients with relapsed spine metastasis and key dosimetric correlations between the risk of spinal cord injury and reirradiation technique, total dose, and time between treatments. The Linear Quadratic (LQ) model was used to convert all the published doses into biologically effective doses and normalize them to EQD2. For 3D radiotherapy, authors used cumulative doses from 55.2 Gy2/2 to 65.5 Gy2/2 EQD2 with no cases of RM mentioned. We found little evidence of RM after SBRT in the papers that met our criteria of inclusion, usually at the median reported dose to critical neural tissue around 93.5 Gy2/2. There is a lack of consistency in reporting the spinal cord dose, which leads to difficulty in pooling data.

## 1. Introduction

In cancer patients, bone is quite a common site of metastatic disease. Involvement of the spine may occur in up to 40% of the cases [1,2]. Survival of these patients varies by type of cancer and extension of disease. Better survival is observed in patients with prostate and breast cancer, with the median ranging from 12 to 33 months, while for patients with lung cancer, only 10–12% will be alive at 12 months [3,4,5], being even lesser for other tumors [6]. Survival is influenced by the time between diagnosis of the primary tumor and secondary bone lesions, timing and number of secondary lesions, and skeletal or extra-skeletal site [4,7,8].

Variable clinical presentations can be expressed in relation to the localization of the vertebral lesion, whether it is found in the bone, epidural space, leptomeninges or spinal cord. There is substantial use of hospital services because of considerable morbidity [9,10,11]. These lesions are often painful and can cause a skeletal-related event, with neurological deficit due to spinal cord compression [12]. Palliation for improved quality of life (QOL) and sometimes curation are obtained with radiotherapy treatments, which play a significant role in management of these lesions [13]. However, one third of the patients that will be alive in one year will experience local relapse. Published data show a 12-month local-recurrence failure rate of 39% for single-dose radiotherapy and 23% for patients treated with fractionated courses [14]. Reirradiation is a viable option for inoperable patients where spinal cord decompression is needed [15,16]. However, as radiation dose to the spine builds up, the risk that one needs to take into account is radiation-induced myelopathy (RM) with severe neurological dysfunction.

Although very rare, RM is a feared complication that, when present, cancels any potential benefit on spine metastasis. To lower this risk, a second local treatment, often in the form of reirradiation, tries to limit the target volume, dose per fraction and field [17]. Endothelial and glial injury, disruption of the blood-spinal cord barrier as well as demyelination are thought to be the underlying mechanisms of RM [18]. Symptoms can vary, and the latent period ranges from a few months to several years following radiation exposure, making the diagnosis difficult [19,20]. Contrast enhancement, spinal cord expansion, atrophy and hyperintense signal changes can appear on T2-weighted MR images. However, these signs are nonspecific, depending on the timing of imaging after radiotherapy, leading to RM to be a diagnosis of exclusion [21,22].

There is a correlation between total dose, dose per fraction and probability of developing complications. The cumulative biologically effective dose (BED) delivered to the spinal cord should be calculated and considered as it predicts the risk of RM [23]. In addition, the influence of other factors was noticed in animal models, such as the time interval to reirradiation, length of irradiated spinal cord and age [15,16].

## 2. Purpose

The aim of this paper is to review the available published data regarding local treatment options after initial irradiation in patients with relapsed spine metastasis, with an emphasis on the risk of spinal cord injury in relation to reirradiation technique, dose, the time between treatments and fractionation. A literature search on PubMed was limited to reirradiation of the vertebral metastases published between 1990 and 2021. Descriptive statistics using Excel were used to summarize the median cumulative dose, median dose per course of radiotherapy, radiation myelopathy cases, dose constraints and current guidelines for reirradiation.

## 3. Methods

We reviewed the most important publications related to vertebral reirradiation. We have included studies that specify the time interval between two treatment cycles, studies that specify data related to overall survival and mean or median follow-up. Our search included all data on adult participants with vertebral tumors, regardless of their gender, race or primary tumor.

Types of irradiation techniques included in our review were 2D and 3D external beam radiotherapy (EBRT), stereotactic body radiotherapy (SBRT), and stereotactic radiosurgery (SRS). We only analyzed papers that specified the technique used in the first and second irradiation, with sufficient dosimetric data available, in the form of dose regimen and fractionation. If certain studies were found to be missing information about median BED or equivalent dose in 2 Gy fractions (EQD2), then we used our own calculations in accordance with the data in the attached tables.

For 2D and 3D irradiation, we assumed that the spinal cord received the total prescribed dose. Instead, for modern techniques that use intensity modulation in volume (VMAT), we collected data related to the maximum dose point and doses in relation to various volumes specified by the authors.

To do the calculations and compare different regimens, we used the linear-quadratic model and its formula: BED = n × d(1 + d/α/β) where n = number of fractions, d = dose per fraction, α = linear component of cell killing, β = quadratic component of cell killing and α/β = dose at which both components are equal [24]. The spinal cord dose was normalized to EQD2, assuming an α/β of 2 Gy.

## 4. Radiotherapy Techniques for the Treatment of Vertebral Metastases

Traditionally 2D and 3D EBRT techniques were used strictly for palliation treatments. Chow’s et al. analysis compared single fraction conformal radiotherapy regimens versus multifractional treatment for uncomplicated bone metastases. The effectiveness of the two is similar, but the need for retreatment after a single fraction was bigger (20%) compared to multifractional treatment (8%) [25]. In our findings, in 3D EBRT, authors used cumulative doses from 55.2 Gy2/2 to 65.5 Gy2/2 EQD2, and we assume that the spinal cord received the full prescribed dose. At these values, no cases of RM were mentioned [26,27]. The median time to local recurrence or progression is about four months, and this may explain the need to escalate the dose and use innovative therapeutic strategies to improve outcomes [28,29].

The inverse planning techniques and intensity modulation using multi-leaf collimators allowed better protection of organs at risk and, implicitly, the spinal cord. Studies conducted by Navarria and Mancosu have demonstrated the feasibility of these techniques. They used conventional dose regimens, but the planning technique used constraints to the spinal canal in order to optimize a donut-shaped isodose so that cumulative dose to spinal canal D1 cc < 60 Gy2/2 [30,31]. The ability to bypass the spinal cord and to apply constraints on certain volumes calls into question the homogeneity in defining the spinal cord and reporting the dose to it. There is uncertainty when delineating and variability in the way delineation of the spinal cord is done. Uncertainty can be minimized by fusioning the planning CT with an MRI, but this also has its limits. Uncertainties related to set-up errors must also be taken into account for more precise dose estimation. There is another option that some use, and that is to evaluate the thecal sac or spinal canal and not the spinal cord. Most of the authors in the analyzed works create a planning organ at risk volume (PRV) margin of 1–2 mm, to which optimization is done.

It is interesting to mention that authors who studied the use of highly conformal radiotherapy techniques such as volumetric modulated arc therapy (VMAT) in reirradiation obtained median doses to the spinal cord of 23.6 Gy2/2 which is a lot better than doses around 40 Gy2/2 obtained with conformal technique in the first course. Using VMAT in the second irradiation offered a median time to local recurrence of nine months [32,33].

Of great importance was the introduction of SBRT 20 years ago. It allowed the escalation of dose while protecting the organs at risk, offering prospects of curability to oligometastatic cases or with typical radio-resistant histology [6]. Also, the immobilization and imaging guidance techniques allowed for higher accuracy and a better safety profile. Work is still needed on the homogeneity of prescribing the doses. Unfortunately, published studies are difficult to compare because of the lack of information related to prescription isodose, which can be of great impact. Compared to conformal techniques, SBRT reirradiation courses that we analyzed offered a median dose to the spinal cord of 26.5 Gy2/2 and permitted a dose escalation on the tumor. Cumulative doses to the spinal cord were comparable to those in the range of safety described for conventional techniques. We found little evidence of RM after SBRT in the papers that met our criteria of inclusion. Ito et al. reported one case, but we have no information on the cumulative doses to that specific patient [34]. In the work of Boyce-Fappiano et al., 2% of the patients developed RM after being retreated with SBRT, one month after the first course of conformal radiation therapy. The median reported dose to critical neural tissue was 93.5 Gy2/2 [35].

Conventional radiotherapy regimens with a conformal 3D technique used a variety of fractionations, but the most common were 1 fraction of 8 Gy, 5 fractions of 20 Gy, and 10 fractions of 30 Gy. On the other hand, SBRT regimens used doses of 16–18 Gy in one fraction, 24–26 Gy in 2 fractions, and 24–27 Gy in 3 fractions. In order to be able to compare all the fractionation regimens, we converted all the doses into biologically effective doses and normalized them to EQD2. For this purpose, the Linear Quadratic (LQ) model was used, although known to have some limitations for doses >10 Gy per fraction. Despite all that, it is still the most used model by authors in SBRT literature, with an α/β of 2 Gy for the spinal cord. The LQ model was also used to transform doses that were reported into 2 Gy per fraction equivalent EQD2 and thus to ease comparison between different plans (see Table 1). The median cumulative dose for the spinal cord in our analysis was 64 Gy (for 3D: 61.5 Gy2/2; for VMAT 65.75 Gy2/2 and for SBRT 64.5 Gy2/2). Local control of pain and disease progression was comparable with the three techniques. Unfortunately, heterogeneity of the histology, stage of the disease and lack of full data reporting make it impossible to compare the analysis for overall survival and efficacy. The use of highly conformal techniques for dose escalation, such as SRS/SBRT, manages to administer higher tumoricidal biologic doses compared to conventional techniques. Tolerance for these regimens seemed good, with less than 2% of grade 3–4 toxicity.

## 5. Questions Regarding the Time Interval between the First and Second Radiotherapy Cycle

The time until reirradiation can give us information about the efficacy of the first course, aggressiveness of the disease, radiosensitivity and the patient’s prognosis. The median time to reirradiation in the papers we analyzed was 14 months. Since most were irradiated in the first phase with conformal techniques and only one author had patients previously treated with SBRT (for Thibault et al. 12.9 months), we cannot draw comparative conclusions regarding median time to reirradiation [36]. Interestingly, for those who reported cumulative EQD2 doses >65 Gy2/2, we found that the median time between the two treatments was 18 months. We believe this larger period of time justifies the assumed risk of increasing the dose.

Preclinical data showed a recovery of damage after a period of more than six months, a benefit that continues one or two years after [37]. In a study conducted by Grosu et al., 3D conformal irradiated patients’ BED ranged from 125–205% of the acceptable BED, with no serious reported toxicities [27]. All the patients died due to the progression of the disease. In the authors’ view, the wide timeframe between the two irradiations correlates with preclinical data on spinal cord recovery and justifies the absence of myelopathy despite the large, administered BEDs. On the same topic, Wong et al. approximate recovery of 10% for <14 months and 25% >14 months between irradiations [15].

## 6. Studies Reporting Radiation-Induced Myelopathy

The main clinical endpoint of this review is radiation-induced myelopathy. The rarity of this diagnosis is reflected not only in common knowledge but also in the very low number of cases found in the literature. The specificity of clinical signs and symptoms is not very high. Many patients already had neurologic deficits before retreatment. Radiological information comes in to help rule out cancer progression, which could create confusion in diagnosis. Clinical judgment is needed for correlation with location, time and dose received by the patient. Another reason for the rarity of this diagnosis is the relatively short survival of these patients, thus lacking a long follow-up and the time needed to develop RM. Table 2 displays the selected studies reporting RM patients.

Wong et al. found 22 cases of myelitis in a series of patients irradiated 2D and 3D. Eleven of them developed RM after the second treatment. Lhermitte syndrome was documented for three patients. Brown Sequard syndrome was also mentioned. Patients experienced neurological symptoms in relation to the reirradiated spinal cord segment. No evidence of the progression of the disease was found either radiologically or microscopically. Diagnosis of RM was confirmed histologically by signs of coagulative necrosis in the white matter, vascular changes, and hyalinization of blood vessels. Patients who underwent just one radiotherapy cycle had a significantly longer latent time to RM (11.4 months) in comparison with those who developed the disease after reirradiation. The median time between the two irradiation cycles was 19 months (minimum of 2 months and maximum of 57 months). Except for one target volume in the cervical segment, all the patients were treated for thoracic vertebrae lesions [15]. The estimated median cumulative EQD2 dose to the spinal cord was 67 Gy2/2.

Five patients reported by Sahgal et al. developed RM after reirradiation of thoracic vertebrae, in the first course being treated with 3D CRT technique to a median EQD2 dose of 38 Gy2/2 and retreated, at a median time of 18 months, with SBRT (median point-maximum-dose to the spinal canal: 61.7 Gy2/2 EQD2) to a cumulative dose of 99.6 Gy2/2. RM was seen at a median latency of five months. Patients developed symptoms such as weakness that progressed to paresthesia, lack of proprioception, paraplegia, and urinary retention. They were classified as grade 4 RM, and their clinical evolution correlated with the reirradiated segments with imaging findings of enhancement and necrosis on MRI [38].

## 7. Surgery vs. Reirradiation

Thibault et al. performed a retrospective study on a cohort of 40 patients in whom 56 spinal metastases were irradiated, and 37 patients underwent surgery before the first or second cycle of radiotherapy. The median time from the first surgery to progression was 11.7 months, and the median time to the second course of SBRT was 1.2 months. In selected patients who were asymptomatic but with radiological progression, time to salvage treatment was prolonged until they became symptomatic, compared to patients with imaging progression and associated symptomatology who underwent immediate decompression surgery [36].

A systematic review of 33 studies described the time benefits of neurosurgery vs. radiotherapy alone. In patients with severe, recently installed neurological impairment, neurosurgery provided the shortest recovery rate for both ambulatory function and pain reduction [39]. However, for operable patients with good overall performance status, the association between spinal cord decompression/vertebral fixation and adjuvant radiotherapy revealed good results in patients with spinal cord compression [40]. Explicitly, reirradiation with SBRT after decompression surgery provided 1-year local control rate of 70% in Ito’s study and up to 88.6% at the time of the last follow-up in a large sample of 426 patients [34,41].

The major advantages of surgery followed by radiotherapy when compared with radiotherapy alone are immediate decompression of the spinal cord and direct mechanical stabilization of the spine. Indications for spinal surgery include intraspinal bony fragment, spinal instability, impending or present sphincter dysfunction, no response to previous radiotherapy treatment, and high-grade metastatic epidural spinal cord compression [42]. Also, surgery followed by SBRT has been shown to be more effective than SBRT alone in the case of bulky epidural metastases because, in this context, the increased size of the target volume would require a lower dose-per-fraction regimen (<10 Gy/fraction) compared to the dose-per-fraction regimen applicable to small metastases (>10 Gy/fraction), the dose-per-fraction being a predictor of local control in the case of SBRT treatment. Furthermore, in cases of bulky spine metastasis, spatial fractionation of high radiation dose allows limiting the intracanal exposure by selectively irradiating with an ablative dose only small tumor subvolumes (i.e., vertices) located away from critical neural tissues. With such an approach, Ferini et al. obtained an almost complete response with long-lasting symptom relief of a bulky gynecological tumor extensively eroding the sacrum until invading the cauda equina through the sacral foramina [43]. The mechanism underlying a similar result likely relies on boosting the host immune response against the tumor thanks to this particular dose delivery pattern [44,45].

Another recent approach is minimally invasive spinal surgery (MISS) with rapid recovery that can benefit from the increased precision of adjuvant SBRT for a higher local control instead of the classic radical surgical resection followed by low-dose conventional radiotherapy [46]. As per Saghal et al., the risk of RM may increase in patients who undergo surgery before the second course of SBRT [38]. Therefore, this combination should be proposed in selected cases (significant epidural disease, symptomatic cord compression or cauda equine syndrome, mechanical instability), taking into account the morbidity associated with surgery.

Prognostic scores are available to guide treatment decisions. A typical multidisciplinary tumor board may find the use of the Rades score to evaluate radiotherapy as palliative treatment for patients with advanced-stage spinal metastasis and Spinal Instability Neoplastic Score (SINS), used especially by neurosurgeons to assess the degree of spinal instability [40].

## 8. Consistency in Delineation and Dose Reporting

There is a lack of consistency in reporting spinal cord dose, which leads to difficulty in pooling data (see Table 3). First, segmentation of the spinal cord can be challenging. Image fusion of MRI or CT myelogram can come in the hand of delineation, while in the absence of better imaging, only the spinal canal can be reliably contoured with the CT alone. Second, most clinicians create a safety margin around the true spinal cord in order to compensate for set-up errors (spinal cord motion is reported to be a submillimeter) [47]. One approach is to apply a uniform margin of (1, 1.5, and 2 mm). Another approach is to define the thecal sac or spinal canal.

Garg et al. reported differences in doses depending on the structures used to specify the dose. For example, in his work, he obtained a median Dmax of 12 Gy to the spinal cord and a median Dmax of 14 Gy to a PRV of 1.5 mm [48].

In order to make comparisons between studies, particular DVH parameters need to be chosen for reporting. The dose specified by a single calculation point, known as Dmax, is the most used. New treatment techniques have required adapted recommendations formulated within the International Commission on Radiation Units and Measurements (ICRU). Therefore, the near-max dose could be less susceptible to uncertainty and a more reliable metric. There is great heterogeneity in the modality of reporting. In most of the studies that were cited, Dmax to a specific structure, usually spinal cord + PRV, was used, although more recent studies using modern techniques tend to apply ICRU recommendations for reporting to a near-max dose or dose to a specific volume. Kawashiro et al. used, for example, D0.5 cc [32].

The issue of volume effects was explored by Sahgal et al., who used 0–1 cc in 0.1 increments, and D2 cc for reporting Dmax. Up to 0.8 cc, there were significant differences between RM cases and controls [49]. Similar volume effects were found by Grimm et al. [50]. All these data are inhomogenous enough to refrain from applying dose-volume constraints. Therefore, recommendations for Dmax to a PRV of the spinal cord are still used in practice.

## 9. Dose Constraints and Prediction of RM

QUANTEC model for conventional fractionation predicts the risk of RM accordingly: Dmax value of 45 Gy: 0.03% risk, 50 Gy: 0.2% risk, 54 Gy 1% risk, and 61 Gy 10% risk. As it is unclear whether the linear quadratic model is reliable in providing an accurate estimation of the EQD2 at high doses per fraction, the QUANTEC should not be used when applying dose constraints for SRS/SBRT.

The work to which most SBRT studies make reference is of Nieder and colleagues. The study retrospectively collected patients with 2D/3D conformal spinal irradiation. Based on the data, we made a prediction score for the RM risk of the second course. This is where the recommendations of cumulative BED <135 Gy, 6-month interval and BED of any course <98 Gy come from [51]. For a single fraction treatment using high-precision techniques such as SBRT, Katsoulakis et al. proposed a Dmax for the spinal cord of 14 Gy, with a risk of RM of 1% [52].

Sahgal et al. reviewed a large cohort of patients with a variety of fractionated de novo SBRT, ranging from 2–5 fractions and proposed the following spinal Dmax constraints: 2 fractions: 17 Gy, 3 fractions: 20.3 Gy, 4 fractions: 23 Gy, 5 fractions: 28.8 Gy with a risk of RM ranging from 1–5% [49]. Table 4 summarizes dose limits for the first course of SBRT. In the case of reirradiation SBRT, the following criteria were taken into consideration: minimum time interval to reirradiation of at least 5 months; cumulative thecal sac EQD2 Dmax <70 Gy; reirradiated thecal sac EQD2 Dmax <25 Gy [38]. Dose limits seen in Table 5 aim to respect the criteria proposed above for reirradiation.

In a study conducted by Hashmi in 2016, patients were retreated with SBRT after conventional EBRT either with a multifractionated regimen (40% of patients) with a median spinal cord Dmax of 15.3 Gy2/2 or a single fraction (60% of patients) with a median spinal cord Dmax of 30 Gy2/2; cumulative BED were 47.8 Gy2/2 and 65.6 Gy2/2, respectively. Although local control was found to be better in single fraction retreatment, in the univariate analysis, this was not correlated with BED. This could be explained by the increased radiobiologic effect of doses >10 Gy discussed above. It is also worth mentioning that lower spinal cord Dmax in the multifractional group was explained by the under dosage of the posterior vertebral body and epidural space [55].

## 10. Conclusions

This review highlights important issues for the reirradiation of patients with vertebral metastases. In our analysis, the median time to reirradiation was 14 months. The use of highly conformal techniques in reirradiation obtained median doses to the spinal cord of 23.6 Gy2/2 versus doses around 40 Gy2/2 obtained with conformal technique in the first course. For 3D radiotherapy, authors used cumulative doses from 55.2 Gy2/2 to 65.5 Gy2/2 EQD2 (or, in some cases, up to 125–205% of the acceptable BED) with no cases of RM mentioned. We found little evidence of RM after SBRT in the papers that met our criteria of inclusion, usually at the median reported dose to critical neural tissue around 93.5 Gy2/2. Radiation retreatment of spinal lesions in the previously irradiated field can be difficult, and radiation-induced myelitis is a serious complication that everyone wants to avoid. Fortunately, high-precision techniques of irradiation can help us where constraints become difficult to achieve and enable dose escalation to gain better tumor control. Despite these advantages, there is a need for greater rigor in terms of the treatment protocol, as there are inhomogeneities in the modeling, prescription and dose reporting. Prognostic scores such as Rades and SINS are available to guide treatment decisions. We need prospective and well-protocolized studies to obtain better-quality results that will ultimately guide us in the effective selection and tailoring of patient retreatment. The ultimate goal is to achieve maximum results in control and survival while minimizing as much as possible the risk of radiation-induced myelitis.

## Figures and Tables

**Table 1:** Technique and dose fractionation for first and second radiotherapy cycle; cumulative doses normalized to EQD2.

Reference Study	Number of Patients	Target Volumes	Median Time to Re-Irradiation (Months)	RT1-Tech	Dose Fractionation (Median Gy/fr)					Dose (Median BED EQD2)				Median Volume Treated (cc/nr. of Vertebrae)	RM
					RT1 DT	RT1 fr	RT2-Tech	RT2 DT	RT2 fr	RT1 (Median EQD2)	RT2 (Median EQD2)	Tumor EQD2-SBRT	Cumulative (RT1 + RT2)		
**Sahgal 2012**	14	16	15	3D CRT	30	17	SBRT	24	3	39.8	12.5		52.4	11.5	0
	5 RM	5	18	3D CRT	40	22	SBRT	20	2	38	61.7		99.6	31.5	5
**Hashmi 2016**	215	247	13.5	3D CRT	30	10	SBRT	18	1	37.5	24.6	36	60.8	No data	0
**Foerster 2018**	16	No data	No data	3D CRT	No data	No data	SBRT	18	1	No data	33.8	41.3	69.9	56.9	0
	7			pSBRT	20.4	1	0	0	0	26	0			81.9	0
**Hirano 2015**	35	52	5.3	3D CRT	30	10	3D CRT	8	1	37.5	20		57.5	No data	0
**Ito 2018**	82	134	No data	3D CRT	25	7.5	SBRT	24	2	30	24	44	54	No data	1
**Zschaeck 2017**	30	31	11	3D CRT = 18	No data	No data	SBRT	No data	No data	33.1	33.5		69	No data	0
**THIBAULT** **2015**	40	56	12.9	SBRT	24	2	SBRT	30	4	31.8	21.9	32.5	51.3	No data	0
		24		3D CRT	No data	No data	SBRT	No data	No data	50.8	21.9		81.4	No data	0
**BOYCE-** **FAPPIANO** **2017**	162	237	10.2	3D CRT	30	10	SBRT	16	1	37.5	56	34.7	93.5	2 v	1
**Mahadevan 2011**	60	81	20	3D CRT	30	10	SBRT	27	4	37.5	30		67.5	84	0
**Choi 2010**	42	51	9	3D CRT	40	20	SBRT	26	2	40	24		64	10.3	4
**Hoyer 2017**	215	247	14	3D CRT	30	10	SBRT	18	1	No data	No data		No data	No data	0
**Navarria 2012**	31		17	3D CRT	30	10	VMAT	30	12	37.5	23.6		61.6	289	0
**kawashiro 2015**	23	23	13	3D CRT	37.5	No data	VMAT	14.5	5	40	17.7		59	47.4	0
**Sterzing 2010**	36		17.5	3D CRT	36.3	No data	VMAT	34.8	No data	40	32.5		72.5	2 v	0
**Folkert 2013**	5	5	12.2	3D CRT	30	10	IOBT	14 Gy	1	37.5	65.3		92.8	No data	0
**Maranzano 2011**	12		6.5	3D CRT	8	1	3D CRT	15	3	30	26		56	No data	0
**Whong 1994**	139		19	2D/3D CRT	24	9	3D CRT	20	8	41	26		67	60	11
**Grosu 2002**	8		30	2D/3D CRT	38	18	3D CRT	30	15	38.5	30		68.5	2	0
**Ahmed 2012**	66	85	13.5	3D CRT	30	10	No data	24	3	-	-		-	42.7	1
**Doi 2021**	32	32	15	3D CRT	30	10		39	13	45.6	80.7		135.6		2 v

**Table 2:** Selected studies reporting radiation induced myelopathy (RM) with corresponding dose and fractionation.

Reference Study		Sahgal 2012		Ito 2018	BOYCE-FAPPIANO 2017	Choi 2010	Whong 1994	Ahmed 2012
Number of patients		14	5 RM	82	162	42	139	66
Target volumes		16	5	134	237	51		85
Median time to re-irradiation (months)		15	18	No data	10.2	9	19	13.5
RT1-tech		3D CRT	3D CRT	3D CRT	3D CRT	3D CRT	2D/3D CRT	3D CRT
Dose fractionation (median Gy/fr)	RT1 DT	30	40	25	30	40	24	30
	RT1 fr	17	22	7.5	10	20	9	10
	RT2-tech	SBRT	SBRT	SBRT	SBRT	SBRT	3D CRT	No data
	RT2 DT	24	20	24	16	26	20	24
	RT2 fr	3	2	2	1	2	8	3
Dose (median BED EQD2)	RT1 (median EQD2)	39.8	38	30	37.5	40	41	-
	RT2 (median EQD2)	12.5	61.7	24	56	24	26	-
	tumor EQD2-SBRT			44	34.7			
	Cumulative (RT1 + RT2)	52.4	99.6	54	93.5	64	67	-
Median volume treated (cc/nr. of vertebrae)		11.5	31.5	No data	2 v	10.3	60	42.7
Clinical end points		Late spinal cord toxicity	Late spinal cord toxicity	Pain relief; local control; adverse events	* Pain response 81% (reduced) * Neurological improvement 82% * Radiographic local control 71%	Local control		Local Control
RM		0	5	1	1	4	11	1
Follow-up (median in months)		12	17	9	4	7		8.2
Spine tumors after re-irradiation (%/m)		57.10%	80.00%	OS 65%/12 months	Median OS = 13 months	24	8.3 m (15% in 5 years)	1 year OS in those with prior RT = 28%
Local Control after re-irradiation		No data	No data	72.3%/12 months	Radiographic local control 71%	74%		1 year LC = 83.3 % with prior RT
Median time to local recurrence or progression		No data	No data	14		8		No data

**Table 3:** Anatomical reference points for dose reporting in selected studies.

Reference Study	Target Volumes	RT1-Tech	Dose Fractionation (Median Gy/fr)					Median Time to Re-Irradiation (Months)	Dose (Median BED EQD2)				REPORT/CONSTRAINTS
			RT1 DT	RT1 fr	RT2-Tech	RT2 DT	RT2 fr		RT1 (Median EQD2)	RT2 (Median EQD2)	Tumor EQD2-SBRT	Cumulative (RT1 + RT2)	
**Sahgal 2012**	16	3D CRT	30	17	SBRT	24	3	15	39.8	12.5		52.4	Dmax to thecal sac
	5	3D CRT	40	22	SBRT	20	2	18	38	61.7		99.6	
**Hashmi 2016**	247	3D CRT	30	10	SBRT	18	1	13.5	37.5	24.6	36	60.8	Spinal cord + PRV 1–2 mm
**Ito 2018**	134	3D CRT	25	7.5	SBRT	24	2	No data	30	24	44	54	MRI spinal cord + PRV 1.5 mm Dmax < 11–12 Gy × 2 fr.
**Zschaeck 2017**	31	3D CRT	No data	No data	SBRT	No data	No data	11	33.1	33.5		69	Spinal canal D50 < 1 cc mean Dmax = 50.8 Gy mean D0.5 cc = 44.9 Gy mean D1 cc = 43.3 Gy
**Thibault** **2015**	56	SBRT	24	2	SBRT	30	4	12.9	31.8	21.9	32.5	51.3	Spinal cord + PRV Pmax PRV
	24	3D CRT	No data	No data	SBRT	No data	No data		50.8	21.9		81.4	
**Kawashiro 2015**	23	3D CRT	37.5	No data	VMAT	14.5	5	13	40	17.7		59	Spinal cord D0.5 cc in reirad 10 Gy/5 fr, cumulated D0.5 cc = 91 Gy 2/2.
**Sterzing 2010**		3D CRT	36.3	No data	VMAT	34.8	No data	17.5	40	32.5		72.5	Dmax in reirad:9.8 Gy (5.2–21.8 Gy) V5 = 6.7 cc; V10 = 2.4 cc, V15 = 0.7 cc

**Table 4:** Dose model-derived limits from publications, for the first course of SBRT, for RM risk of 1–5%.

Number of Fractions	Recommendations for Thecal Sac/Spinal Cord Dmax (Gy)			
	AAPM TG101 [53]	Kim 2017 [54]	Sahgal 2013 [49]	Katsoulakis-Gibbs [52]
1	14	14	12.4	14
2		18.3	17	19.3
3	21.9	22.5	20.3	23.1
4		25.6	23	26.2
5	30	28	25.3	28.8

**Table 5:** Recommendations for reirradiation, taking into account prior conventional EBRT–Maximal proposed spinal cord Dmax was derived from criteria associated with no RM case in Sahgal’s study [38].

		Dmax (Gy)				
Prior Dose Fractionation	EQD2 (Gy)	1 Fraction	2 Fractions	3 Fractions	4 Fractions	5 Fractions
20/5	30	9	12.2	14.5	16.2	18
30/10	37.5	9	12.2	14.5	16.2	19
40/20	40	-	12.2	14.5	16.2	20
45/25	43	-	12.2	14.5	16.2	21
50/25	50	-	11	12.5	14	15.5

## Data Availability

Not applicable.
